# Enacting biosocial complexity: Stress, epigenetic biomarkers and the tools of postgenomics

**DOI:** 10.1177/03063127231222613

**Published:** 2024-01-12

**Authors:** Luca Chiapperino

**Affiliations:** University of Lausanne, Lausanne, Switzerland

**Keywords:** epigenetics, postgenomics, biosocial, biopsychosocial, material-semiotics, stress, complexity, complexities, complexification

## Abstract

This article analyses attempts to enact complexity in postgenomic experimentations using the case of epigenetic research on biomarkers of psychosocial stress. Enacting complexity in this research means dissecting multiple so-called biosocial processes of health differentiation in the face of stressful experiences. To characterize enactments of biosocial complexity, the article develops the concepts of *complexity work* and *complexification*. The former emphasizes the social, technical, and material work that goes into the production of mixed biological and social representations of stress in epigenetics. The latter underlines how complexity can be assembled differently across distinct configurations of experimental work. Specifically, complexification can be defined as producing, stabilizing, and normalizing novel experimental systems that are supposed to improve techno-scientific enactments of complexity. In the case of epigenetics, complexification entails a reconfiguration of postgenomic experimental systems in ways that some actors deem ‘better’ at enacting health as a biosocial process. This study of complexity work and complexification shows that biosocial complexity is hardly a univocal enterprise in epigenetics. Consequently, the article calls for abandoning analysis of these research practices using clear-cut dichotomies of reductionism vs. holism, as well as simplicity vs. complexity. More broadly, the article suggests the relevance of a sociology of complexification for STS approaches to complexity in scientific practices. Complementing the existing focus on complexity as instrumental rhetoric in contemporary sciences, complexification directs analytical attention to the pragmatic opportunities that alternative (biosocial) complexities offer to collective, societal, and political thinking about science in society.

Epigenetics lacks a clear-cut definition (Griffiths & Stotz, 2013; Meloni & Testa, 2014; Stotz & Griffiths, 2016). Some define it narrowly as the study of modifications of DNA, RNA, and chromatin that change genome expression without altering DNA sequence (Bird, 2007). Others define the term broadly as the study of ‘the causal mechanisms by which genotypes give rise to phenotypes’ (Griffiths & Stotz, 2013, p. 112). Far from finding a resolution (Horsthemke, 2022), these theoretical disagreements structure heterogeneous strands of research that only partially overlap with one another (Larregue et al., 2020). Disagreements notwithstanding, some see epigenetics as the study of the emergent properties in the origin of organismic phenotypes (Hall, 1992). The epigenome gets described (alternatively) as the mechanism of genomic expression in cellular differentiation and reproduction, as a driver of development and inheritance, or as the interface in gene-environment interplays. Epigenetics (in its different forms) is often taken to bring life scientists ‘closer to a more realistic *understanding of life’s complexity and diversity*’ (Badyaev & Uller, 2009, p. 1169, emphasis added).

Richardson and Stevens (2015) remind us that the resurgence of complexity in epigenetics makes it an ‘archetypal postgenomic science’ (p. 4). Although postgenomics is itself an ambiguous term (Griffiths & Stotz, 2013; Morange, 2006), I believe this is an apt description for two reasons. First, it encapsulates how epigenetic research departs from a closed, simplistic and gene-centric conception of biology and health. The complex interactions (e.g. biological–social, systemic–genomic, or genetic–environmental) at the center of its agenda move it beyond a strong emphasis—dominant before the Human Genome Project (HGP)—on genes and their molecular characteristics (Griffiths & Stotz, 2013). Epigenetics is thus *post*-genomic because it offers complex biological facts that move past genes as central explananda of life and health. Second, epigenetics can be called post-*genomic* with the opposite emphasis. Critics have in fact questioned whether the alleged biological complexity enacted in epigenetics really does break with pre-HGP life sciences. In this view, epigenetics does nothing more than revive historical tropes of complexity, plasticity, and environmental thinking in biology from the material substrates, tools, and experimental systems established with the HGP (Morange, 2018; Rheinberger & Müller-Wille, 2018). Far from neutral, these technoscientific conditions of possibility of complex epigenetic facts should make us wary of any ‘feeling of radical novelty’ (Morange 2018, p. 189). Neither of these themes are specific to post-HGP life sciences—including modern epigenetics (Meloni, 2019; Peterson, 2017). Nor have the reductionist, gene-centric and deterministic explanations of molecular biology ‘disappeared or been dramatically transformed’ (Morange, 2018, p. 190). In this view, epigenetics does nothing but affirm the centrality of the tools, styles of reasoning and explanations of genomics in refreshing an old interest in the complexity of living beings.

These epistemological tensions around complexity in postgenomics have attracted substantive scrutiny (Griffiths & Stotz, 2013, ch. 5; Morange, 2006; Richardson & Stevens, 2015). As Dan-Cohen argues, assessments cluster into a ‘glass-half-empty’ versus ‘glass-half-full’ logic (Dan-Cohen, 2016, p. 908). Some emphasize the gaps between complexity as enacted in scientific work and the complexity of living beings. For others, the very resurgence of attention to complexity among molecular biologists is a significant shift to record. Still others underline the *longue durée* of the tensions between reductionism and holism, or between determinism and emergence in biological knowledge production (Meloni, 2016, 2019; Morange, 2018; Rheinberger & Müller-Wille, 2018). If anything, the tightrope ‘at the fuzzy boundary *between* the trivial and the complex’ is a major defining feature of biology’s experimental systems since their emergence (Rheinberger, 1997, p. S247, original emphasis). Within this literature, less attention has been devoted to the specific ‘facts and processes’ that occur in the contemporary laboratory when ‘complexity is at the tip of so many tongues’ and, at the same time, unsettles the epistemological tenets of a field (Nelson, 2018, p. 209). In other words, fewer have asked: what do distinct attempts to enact biological, environmental, and psycho-social complexity look like in practice?

Integrating analyses of postgenomics with studies of complexity in science and technology studies (STS), this article offers one answer to this question. It describes how scientists navigate the unstable epistemic space of complexity in postgenomics. It does so in a specific sub-field of epigenetics investigating the biomarkers of psychosocial stress (Sandi & Haller, 2015). While terms such as ‘complexity,’ ‘complex,’ or ‘complex system’ are difficult to define even for complexity scientists (Parisi, 2007), in my fieldwork these notions typically referred to the process of folding a biological and social understanding of stress-related diseases together into the epigenome. Epigenetic phenomena therefore qualify as ‘complex’ among my informants in the sense that multiple so-called biosocial (e.g. social-to-biological, biological-to-social, or even social-in-biological and biological-in-social) processes produce them.^1^ In studying biosocial complexity as an enactment, this article develops an intuition from material-semiotic work in STS. Material-semiotic approaches in STS assume that there is no single reality to be captured and shaped by the material (e.g. technological, organic) and semiotic (e.g. relational, meaning-making) networks of scientific practice. Heterogeneous practice*s* weave the realit*ies* of scientific objects; they perform the relations that make facts and objects circulate among actors. Transposing this intuition to my fieldwork allows me to resist the idea that epigenetic research either fails or succeeds in enacting a single order of biosocial complexity. Rather, the material-semiotic research question of this article is one of multiplicity: how do epigenetic scientists enact biosocial ‘complexit*ies* in practice*s*’? (Law & Mol, 2002, p. 6 original emphasis).

To distinguish among these practices, I employ the idiom of *complexity work* and *complexification*. What I call complexity work emphasizes the social, technical, and material work that goes into the production of a mixed biological and social representation of stress in epigenetics. From this perspective, complexity is not just talked about differently by the actors I encountered.^2^ Rather, it is assembled differently in ways that can: (1) question simplistic representations of biology as isolated from the environment, (2) call into question the representations of these biosocial processes as linear gene-environment interactions, and (3) embrace a dynamic, multi-layered approach to the biosocial processes of coping with stress. My contention is that this spectrum of complexities rests upon an ordered set of material-semiotic assemblages (e.g. research designs, tools, ideas, relations, collaborations, etc.) that enable the enactment of biosocial entanglements of stress in experimentation (Law, 2009). Complexity work is therefore a formula I do not employ to qualify the enactment of a specific kind of biological complexity. Taking performativity and multiplicity seriously, complexity work instead designates *any* research practice actors undertake to connect (components of) the organism with (components of) its material and social environments with the aim of enacting complexity. If an instance of epigenetic research is declaredly a science of the interrelatedness of the biological, psychological, environmental, and social determinants of health, then it is one form of complexity work.

This performative way of thinking also suggests that biosocial complexities can be enacted differently. As Morange (2006) has argued, postgenomics has (again) shifted the pendulum towards the need to explore new approaches to ‘the complexity of feedback and crosstalk’ in biology, or to the ‘high number of components’ of disease (pp. 356–357). Thus, an exigency emerges to produce new ideas, tools, and knowledge (i.e. new material-semiotic assemblages) that can ‘better’ capture the multidimensional, biological-and-social processes producing health. Taking the case of stress in epigenetics, I identify this distinct kind of complexity work as *complexification*. Complexification can be defined as producing, stabilizing, and normalizing novel experimental systems (e.g. research designs, methods, techniques) that are supposed to improve—in the view of the concerned actors—techno-scientific enactments of complexity. The term has been seldom used in STS studies of complexity. Some employ it to describe the normative work that STSers do in engagements with biomedical actors (Zuiderent-Jerak, 2010). For others, the term captures a much-needed switch from a simplistic to a multidimensional framing of public policy issues such as food security, the health effects of obesity, abortion, or the COVID-19 pandemic (e.g. Cardon, 2020; Clarke & Montini, 1993; Ulijaszek, 2015; Wagenaar & Prainsack, 2021). While certainly akin to the use I make of the term, these previous contributions on complexification emphasize more the normative work of complexity thinking than the technoscientific and material-semiotic work that makes the knowledge base for policy change.

My inquiry into complexity work and complexification follows in two parts. The first explores ‘complexification’ in contrast to an archetypal postgenomic science. Enacting postgenomics—in the field of social epigenetics—is hardly a univocal enterprise. This endeavor relies on a distinct form of sociotechnical work (i.e. complexification) that re-interrogates the tools of the field to enable alternative representations of how ‘the social’ intertwines with ‘the biological.’ Such a diversity of approaches calls for further studies of the situated enactments of biosocial complexity in experimentation, and for a stronger focus on diversity and heterogeneity in STS studies of postgenomics. So far, critical empirical assessments of these practices have contrasted the biosocial facts produced in epigenetic research with a thick conception of biosocial complexity (e.g. Chiapperino, 2021; Lappé, 2018; Niewöhner, 2011; Penkler, 2022). As a complement to this approach, the present article calls for interrogating the pragmatic opportunities that alternative constructions of biosocial facts in epigenetics offer to collective, societal, and political thinking about this knowledge. Is every biosocial fact in the postgenomic lab the same, even if it fails to mark a qualitative shift from simplicity to complexity, from thinness to thickness?

Secondly, and more importantly, my argument suggests the relevance of a sociology of complexification for STS approaches to complexity in scientific practices. Some STS research points to the need for abandoning categories such as ‘simplification’ (Law & Mol, 2002, p. 4) or ‘reductionism’ (Nelson, 2018, pp. 201–209) to make sense of scientists’ ways of constructing complex scientific facts. Yet, oftentimes, STS critique also dismisses the ‘flourishing complexity discourse’ (Dan-Cohen, 2016, p. 902) in contemporary sciences as rhetoric, or as a way to merely perform anticipatory, reputational and interpretational work. While keeping these suspicions alive is important, I contend that they risk missing out on the situatedness of complexity work done by the actors. The scientists I followed navigated an ontologically productive tension. As an analytical approach, a sociology of complexification underlines the importance of studying such enactments as material-semiotic practices. While consisting of (marginal) adjustments to simplistic/reductionist and mechanistic ways of knowledge-making, these practices enable scientists to coherently demarcate alternative constructions of (biosocial) facts. In the discussion, I elaborate on the reasons why this is ‘a difference worth making’ for the sake of policy-related and collective thinking about the biosocial in society (Vogel & Mol, 2014, p. 315).

## Social studies of complexity

In STS, complexity has been described as a rhetorical/argumentative device to explain past failures and nourish promises (Arribas-Ayllon et al., 2010). Alternatively, it has been shown as having little effect on the technologies and research programs of scientists (Panofsky, 2015). Writ large, complexity in STS is *talk*: it is a discursive practice that performs anticipatory work on the future of a discipline; it explains a field’s challenges; it contextualizes facts being produced, etc. Some STS studies have divided complexity into distinct epistemic regimes of knowledge-production (Li Vigni, 2021). This approach points to the epistemological assumptions and socio-material configurations of scientific research that produce complexity. For instance, in her book *Model Behavior* (2018), Nelson offers an extended examination of the trials and challenges, if not the ‘crisis’ (p. 43), faced by scientists studying the complexity of behaviours and their genetics with laboratory animals. More than enacting a specific philosophical theory of complexity, or capturing its reality, the work of these scientists uses narratives of complexity to shape scientific expectations, experimental configurations, and justify standards of knowledge production (Nelson, 2018). In a similar vein, Levin (2014) has shown how metabolic complexity is not discovered but ‘dynamically entangled’ with methodological innovations in omics science (p. 570). Folding complex representations of the metabolome into computational biology methods, Levin argues, allows scientists to move beyond a simplistic view of biological causation. Similarly, Dan-Cohen (2016) characterizes as ‘epistemic complexity’ the concrete strategies for constructing evidential claims of complexity in life sciences research. She argues that these practices of knowledge production do not necessarily set out to seize the ‘ontological complexity’ putatively ‘independent of knowers and their models, theories, or analytics’ (p.903). Rather, complexity should be taken as epistemic artifact—present in so-called hard and soft sciences (Dan-Cohen, 2017)—that is meant to produce facts and claims giving the impression that ‘the world is a complex place’ (Dan-Cohen, 2016, p. 903). These practices of epistemic complexity, she argues, constitute methodological tactics taming complexity in scientific practice. Complexity therefore is not just talk, but more a tool to combat the liability of a field, establish expertise and normalize methods than it is an effort to portray an alleged reality ‘out there.’

Few studies have focused on how these different ‘layers of complexity’ get folded into epigenetic research (Chiapperino, 2021, p. 46). As pointed out by Penkler (2022), a complex version of postgenomics is always present in the talk of epigenetic scientists; that is, in their frustrations, explanations, expectations, as well as aspirations. Lloyd and Raikhel (2015) have pointed to complexity enacted through experiments in epigenetics and contrasted it with the credibility of the field. The biosocial facts made in the epigenetic lab, and their credibility, result from a ‘complex terrain’ made of ‘technical factors, the state of knowledge, career plans, sample sizes, renown, and politics at large’ (2015, p. 739). In a series of articles (2011, 2020), Niewöhner has formalized this pragmatic approach to the complex ‘entanglement of nature and culture’ in epigenetics (2020, p. 50). This field exemplifies well how complexity is far from being a fact or a discovery of experimentation, but is rather a pragmatic reduction of the complex intricacies of ‘a human biology embedded in social environments’ to functional units of experimentation (e.g. questionnaires, or simple metrics) manageable by the average lab (2020, p. 53). Scientists’ complexity work is not, in other words, guided by the intent of capturing the complex biosocial reality of an organism (Niewöhner, 2020, p. 53). Rather, considerations of opportunity, feasibility and scalability of their experimental possibilities drive the technoscientific work capturing the biological correlates of ‘social differentiation’ (2020, p. 52).

In the remaining sections, I develop these views on complexity as talk, tool, or pragmatic enactment shaped by social and rhetorical factors. My aim is to single out the ‘collateral realities’ (Law, 2009) woven into the technical work of experimenting with complexity. In particular, I am interested in the experimental machineries of epigenetics that assemble different realities (ontologies) of the biosocial. Distinct configurations of tools and research designs in epigenetics mean assembling novel patterns of biosocial relations among experiences and biology. And while the elements of rhetoric and convenience are certainly present in these practices (as I detail below), I also ask a different question: how do these practices recast biosocial processes and the representations of social-and-biological modulators of health? And how is this difference instructive of a different circulation of biosocial facts in society?

## Materials and methods

This article draws from interview data (N = 38), literature analyses, and observations from five years of fieldwork documenting the production of epigenetic knowledge for uses in public health, biomedical research, and policymaking. The interviewees are based in different countries: Australia, France, Italy, Spain, Sweden, Switzerland, The Netherlands, United States, United Kingdom.^3^ They span across different academic positions, although the sample contains more Principal Investigators (N = 29) than post-doctoral and research fellows (N = 9), and more biologists (N = 21) than medical doctors (N = 13) or other backgrounds (N = 4). Most of these informants work in sub-fields of epigenetics that are concerned with the ‘social’ epigenome (Deichmann, 2020). These include a heterogeneous set of experimental studies of the epigenetic effects of acute and/or chronic stress in animal models and/or humans (Sandi & Haller, 2015), which I will discuss in the next section. Imbued with the biosocial questions attracting much STS attention, these practices may not be representative of all the ways epigenetic researchers enact complexity in other corners of the discipline (Deichmann, 2020). Yet, observing them offered the opportunity to analyse how ‘the social’ and ‘the biological’ are drawn together through research practices, meetings and public events in social epigenetic research.

## Letting psychosocial stress flow into experimentation

Cantor and Ramsden (2014) have argued that the diachronic variety of stress is two-dimensional. On the one hand, stress mutated its cultural and social functions since its conventional birth in the 1930s (Jackson, 2014). The practices and concepts associated with stress spread over a heterogeneous epistemic space throughout the 20^th^ century: stress was studied in bodies and populations, in laboratory and field sciences, in relation to physiology and behaviour, as an animal and human phenomenon, as a normal and pathological attribute. Stress came into the limelight of major diseases in the 1940s; it moved back and forth from the epistemic space of an exceptional disease (e.g. related to trauma and collective events, especially in its standardized diagnostic version as post-traumatic stress disorder, or PTSD^4^) to that of an ordinary condition of the modern world (e.g. relating to social conditions and economic precarity).

This section explores how this diachronic variety of stress can be found in its synchronic deployment as an epistemic object of research among a sample of epigenetic researchers.^5^ Stress is, in fact, a polysemic object of experimentation among my informants. Some study it as a chronic condition. Stress means long-lasting childhood adversities posing a developmental threat to adult mental health (e.g. Park et al., 2019). Or, it means lifelong psychosocial adversity due to socio-economic conditions affecting individuals (and their biology) unequally (e.g. Geronimus, 2013). Others study stress as an acute phenomenon, which often borders on trauma (e.g. Vinkers et al., 2015). To these informants, stress is a singular, disruptive event that becomes an etiological factor (via epigenetic mechanisms) in clinical manifestations such as PTSD or major depressive disorder (e.g. Vinkers et al., 2015). But for all these approaches, stress is an exemplary postgenomic object: it is a prominent non-genomic factor involved in development, life course health trajectories, or (to some) inheritance (e.g. Robinson, 2018). The types of acute stressors studied by the informants are related to important social issues in family relations, such as conjugal violence (e.g. Schechter et al., 2015), or collective events (e.g. military service, war-related traumas) whose cultural and social significance is urgent (e.g. Lehrner & Yehuda, 2018). The informants study stress (in its various declensions just mentioned) in animal models and/or in humans, with substantive overlaps between these two communities through translational research (e.g. Turecki & Meaney, 2016). This work is often directed at the discovery of putative biomarkers of psychosocial factors and stressful environments (e.g. Yousefi et al., 2022), even in the case of fundamental research in psychiatry (e.g. Coda & Gräff, 2020). In the face of such a diversity of approaches, the reader will not be surprised at the lack of consensus among interviewees regarding how to apprehend stress as a psychological and social phenomenon. As one informant poignantly replied to a question on measures of ‘psychosocial stress’: every strand of research, if not ‘every lab[,] tries to grasp the most of this complexity in its own way’ (Interview Cécile, Neuroscientist and Psychiatrist).

Yet, lack of a common definition for psychosocial stress does not necessarily entail a lack of shared methodological approaches. Three families of tools for stress measurement regularly came up in interviews within the context of human studies.^6^ The first cluster consists of methods for the measurement of stress *exposures*. Taking the form of interviews or questionnaires, these methods assess whether the respondents experienced a given set of stressful life events. These tools inquire both into acute life events (e.g. adverse childhood experiences) and chronic difficulties (e.g. financial precarity). For each stressor, they entail exploring the context of the stressor (e.g. age at exposure, duration, and severity). A second cluster of tools focuses on perception and aims at measuring the impact of the stressor on the individual; that is, their physical and psychological *response*. Stress responses of this kind are often measured through self-reported questionnaires or interviews, emphasizing the subjective character of stress. Stress responses span from emotional (e.g. frequency of feelings of anger) to cognitive (e.g. difficulty envisaging how to cope with one’s life circumstances) and behavioural (e.g. lack of control of feelings or actions) changes following acute stressful experiences. The Perceived Stress Scale (Cohen et al., 1983) is a 10-question self-reported measure of this kind that interviewees frequently referenced. Finally, the third set of methods sits in between an exclusive focus on the stressor and the stress response, and is defined by a *clinical* focus (Pai et al., 2017). Informants used DSM-based diagnostic criteria to assess stress through symptoms, such as arousal, re-experiencing, or intrusive images. These tools resemble the second cluster but treat response as the manifestation of clinically relevant symptoms. These methods also attempt to quantify stressors as (what the actors call) ‘traumatic load’ (Interview Joseph, Neuroscientist); that is, the number of different traumatic events witnessed and their type (e.g. witnessing of killing, or being abducted).

This taxonomy of mainstream methods for apprehending stressful experiences in epigenetics is perhaps far from exhaustive. This was in fact a strikingly difficult part of my research. Questionnaires, surveys, self-reported measures, and diagnostic assessments are often neglected elements of the epistemic configurations of stress epigenetics. Or, at least, they are a set of tools which laboratory scientists have little familiarity with: ‘you should ask the study nurse’, or ‘you should ask the details to the epidemiologist/clinician in charge of the cohort’ were common answers from interviewees. Collecting these data is a task that interviewees treated as outside the realm of laboratory work. This ‘is not epigenetics’ (Interview Marie, Neuroepigeneticist); these are ‘classic’ instruments, ‘standard’ questionnaires, ‘conventional diagnostics’—or, blackboxed tools (Latour, 1999)—imported from neighboring psychological sciences. Even when employed by lab members, these instruments are not considered as internal to the trials, errors, and adjustments of the lab. Thus, their role in constraining the production of a complex biosocial understanding of stress is seldom discussed. And yet, these tools are central for doing a specific form of complexity work: they provide a numerical measure of stress as experience, a ‘validated’ score that can be used in correlation with quantitative measures of biological differences. More than just performing the complexity work of grasping ‘the most of [biosocial] complexity in its own way’ (Interview Cécile, Neuroscientist and Psychiatrist), each of these instruments enacts a typical epistemic virtue of biomedical sciences: quantification as a laudable trait of knowledge practices (e.g. a mark of objectivity) and scientific attitude (e.g. precision) (Daston, 1995).

**Figure 1.**
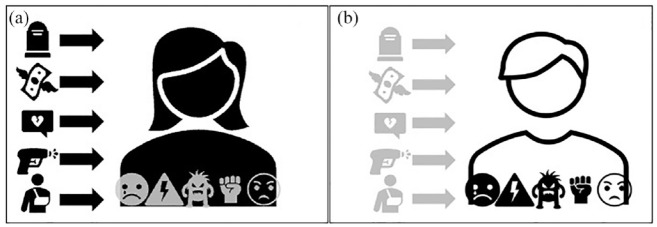
A basic, linear and hierarchical representation of psychosocial stress. (a) Stress as *stressor* (icons in black) linearly flows into the body and is a set of defined psychosocial entities (e.g. traumatic experiences, violence adverse childhood), which are independent from individual’s embodiment (icons in grey) of stress. (b) Stress as *response* (icons in black) is a set of measurable psychological or physiological parameters (e.g. behavior change, feelings) detached from life histories, specific events or social circumstances (icons in grey). Both types of stress can also be measured through instruments focusing on clinical diagnosis.

Second, and relatedly, these customary tools re-produce a view of the entanglements between stressors, experiences, and stress responses, which is *basic, linear, and hierarchical* (Figure 1). It is basic because stress is—as seen through these tools—a self-reported, visible, or conscious phenomenon. What counts as stress can only be whatever ‘objective’ assessment of a traumatic experience can be gleaned from a diagnostic tool. Put differently, stress counts only insofar as it is captured by the probe measuring it: it is either an identifiable environmental factor independent from the embodied experience of the situation (i.e. stressor) or a psychological response independent from life contexts (i.e. response). These tools also commit to a linear model of causation. Stress and related disorders (or biological modifications) originate in a defined stressful event (or series of events) and end with a response or pathology. The relationship between the stressor and the response is unidirectional: the two are separate entities that do not co-produce one another. Finally, these models are also hierarchical in that they implicitly affirm that the only stress that matters is the one that can be detected by allegedly objective indicators. The pre-defined list of exposures or responses/symptoms prevents an appreciation of the individual’s unique embodiment of this stressor in life histories and social circumstances (for a global critique of stress measurements, see Epel et al., 2018).

## Complexifying psycho-social stress in experimentation

Some informants affirm the need to move past these standard measures. As we shall see in this section, a specific kind of sociotechnical work—which I describe as complexification—ensues from these critical stances.

To Cécile, a neuroscientist and clinical psychiatrist, mainstream instruments for measuring stress leave out a lot. For instance, they do not account for timescales, multiple interactions of effects (e.g. relational, psychological, biological), and chronicity of stress. Often, psychosocial stress ‘does not have a defined beginning or end’, thus it cannot ‘really’ be measured as stressor or exposure. When understood in a life course perspective, such as financial precarity, another problem arises: current methods and research designs prevent scientists from studying the additive or interactive role of different stressors (e.g. financial precarity plus the trauma of a life event). The ‘fact of the matter,’ Cécile argues, is that ‘this complexity can hardly be grasped in an objective way through most of these questionnaires alone.’ Dealing with the ‘patient as a whole,’ she claims, is something left to clinical practice. While Cécile, as a systemician psychiatrist (*psychiatre systémicienne*), thinks ‘there is no psychiatric disease without a context,’ it is another issue whether her ‘methods of translational neurosciences’ can reconcile biological markers with such a systemic approach (Interview Cécile, Neuroscientist and Psychiatrist). Roughly put, the way she apprehends stress as a psychiatrist does not correspond to the ways she produces stress as an experimental neuroscientist.

Cécile here offers a rationale for a complexification of stress epigenetics: this would consist of rewiring the tools of this science to apprehend the interactions of social, psychological, and biological factors of stress. A practical answer is offered by Bertil, a rehabilitation researcher who turned to epigenetics to develop biomarkers (combined with clinical and psychosocial data) predicting hospital outcomes of patients affected by chronic pain. Many of the guidelines in pain studies, he elaborates, are ‘based on a biopsychosocial approach’ and measures of the psychosocial abound in his field. However, these measures often ‘lack objectivity’ and are ‘remarkably poor on the bio- side of biopsychosocial.’ Too few studies, according to Bertil, account for the additive and interactive role of different stressors and individual biological predispositions. In a recent article, he and his colleagues found that patients affected by chronic pain due to orthopedic trauma displayed an epigenetic modification of the BDNF gene compared with controls. As Bertil explains, BDNF is known to be important for neuroplasticity and pain sensitization. Methylation of its promoter might be ‘a feedback mechanism, appearing when pain turns chronic, in order to reduce it on the long term;’ so, he underlines, the epigenetic downregulation of BDNF (due to the methylation of its promoter) could be a biomarker to parse out good prognosis patients (Interview Bertil, Biologist).

Bertil and colleagues’ data show that patients with consistently higher levels of pain and worse hospital trajectories have lower average methylation of BDNF (and therefore higher expression of the gene). Yet, these biological differences cannot be taken as stand-alone predictors of the patient’s trajectory. There is something ‘well-known to rehabilitation researchers,’ Bertil elaborates, which ‘heavily impacts’ the level of pain and its biology regardless of the severity of the injury. This is what he and colleagues call patient ‘biopsychosocial complexity’ (BPS complexity) or ‘psychosocial co-morbidities;’ that is, the numerous social circumstances, co-occurring stressors, co-morbidities, or psychiatric conditions that affect the outcome of somatic diseases and patient hospital trajectories. Thus, he concludes:

With this study, I told my colleagues ‘let’s go fishing.’ We have so many data on patient experience: PROMs [Patient-reported outcomes], questionnaires, Fear-Avoidance Belief tests, etc. So, I asked: what if we shifted the focus from the injury—and the sizable stress related to it—to a measure of patient psychosocial complexity that, in combination with a biomarker, can predict hospitalization? We are away from the real world, measures of experience are complicated, but with the COMPRECARE questionnaire we could at least score such complexity and use it for correlations with biological data. (Interview Bertil, Biologist)

To shift from a granular focus on stress (i.e. as either injury or pain), Bertil and colleagues use the COMPRECARE (Comprehensive Care) questionnaire.^7^ This is a clinician-rated tool (based on semi-structured interviews) that is said to operationalize Engel’s biopsychosocial model of disease and integrate psychosocial co-morbidities in one measure (Engel, 1977).^8^ It contains interview items clustered in four domains (biological, psychological, social, and healthcare-related), which are assessed over past, present, and future time. Each question is scored by the interviewer, generating a total score that stands for a measure of so-called BPS complexity. It is one of ‘the most strongly validated tools’ used to assess ‘the BPS complexity of patients,’ with ‘multiple translations and applications throughout the world’ (Interview Bertil, Biologist). In their study, Bertil and his colleagues show that the COMPRECARE score inversely correlates with average methylation values of BDNF and with rehabilitation outcomes. The higher the BPS score (as measured by COMPRECARE), the lower the methylation of BDNF and therefore the higher the level of transcription of this gene. This upregulation of BDNF is, in turn, what produces a higher sensitization to pain and may therefore be implicated in a worse hospital outcome. The ‘precise mechanism’ is unknown to Bertil and the study co-authors, but their results suggest that ‘BPS complexity’—and not just a simple ‘stressor like the injury, or the perceived chronic pain’—heightens pain severity in synergy with BDNF expression. The COMPRECARE score declaredly ‘allows [them] to differentiate the psychosocial factors that modulate levels of BDNF’ and contribute to ‘a better or worse patient evolution’ (Interview Bertil, Biologist).

**Figure 2.**
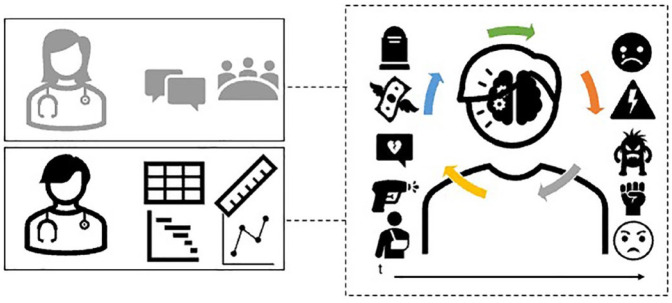
A dynamic, multilevel and systemic view of psychosocial stress. Stress assembled as biopsychosocial complexity (right box) extends over time and emerges from the interaction of social circumstances, co-occurring stressors, co-morbidities, etc. Different approaches to enact this view are possible: for instance, in the context of the doctor-patient relationship (top left box), or through instruments that are amenable to the experimental configuration of the epigenetics lab (bottom left box). The latter, which constitutes a practical and methodological reconfiguration of the experimental systems of epigenetics, is a case of what I call ‘complexification’.

Much like Cécile, Bertil and his colleagues criticize the basic and linear understanding of stress provided by common tools and research designs of the field. Stress can neither be simply objectivized once and for all as stressor or trauma (e.g. the injury), nor can it be easily measured as distinguishable individual responses (e.g. pain). It is best understood within a broader process, which extends into a patient’s biographical events, socioeconomic conditions, and co-morbidities. Studying ‘the patient as a whole’ (Cécile), or ‘biopsychosocial complexity’ (Bertil) reassembles stress (and its effects over health) differently from the questionnaires we encountered in the previous section. Specifically, this configuration draws attention to the dynamic, multilevel, and systemic aspects of this phenomenon (Figure 2): the stressor generates its effects within the context of a patient’s biological, psychological, and social conditions. Where Cécile and Bertil differ is in the enactment of this view. Cécile’s uptake of this view of patient complexity is a function of a discursive—perhaps rhetorical, but certainly relational—configuration that is the doctor-patient relationship. Bertil’s view of stress finds instead a thoroughly different practical enactment, which qualifies as (what I here call) complexification of his experimental systems. This consists of redrawing the network of tools of epigenetics by connecting them with psychosocial measures borrowed from neighboring medical fields. This coherently articulates a *different* relation between the biopsychosocial dimensions of stress. The web of materials, knowledge, practices, and tools assembled by Bertil and colleagues has, in material-semiotic terms, a thoroughly performative effect (Law, 2009; Law & Mol, 2002). This is not meant to deny that it is also a simplification of an alleged ontological thick biosocial complexity. Bertil’s methodological innovation is in fact limited in many ways: it is far ‘from the real world’ (i.e. an alleged ontic reality of this complexity), as Bertil himself concedes. In other words, it is far from exhaustively capturing the messiness and complexity of social-and-biological modulators of chronic pain. One may add that these ways of producing biosocial complexity may still be incommensurable with those purported in STS thinking (Bieler & Niewöhner, 2018; Chiapperino, 2023). While Bertil’s work affirms the importance of social factors and biopsychosocial complexity, it only lets a specific understanding of ‘the social’—one amenable to measurement and correlation (through the COMPRECARE questionnaire)—flow into epigenetic research.

Yet, my point is about what one can learn from studying the multiplicity of these ‘modes of ordering’ biosocial complexit*ies* (Law & Mol, 2002, p. 11). These practices usefully illustrate the kind of work scientists must perform to navigate the epistemic space of stress research in postgenomics. Currently, the complexity work accessible through the common tools for stress measurement is heavily challenged. To enact a view of stress as a biopsychosocial process, scientists must complexify their practice: they must re-order tools, and re-design their studies in ways that can integrate different personal data, stressful experiences, and psychiatric co-morbidities (as well as disease biomarkers; see the following sections). As limited as these practices may be, my argument is that they reveal something about these epistemic processes. Doing postgenomic research on stress still consists of a reductionist task to decompose complex phenomena into relevant causal factors. Yet, the *quality* of this work also depends on the equally important task of *recomposing the unity and multiple dimensions of* biopsychosocial complexity within an experimental system. A postgenomic science of stress cannot, in other words, function independently from a complexification of the tools to restore its biopsychosocial dimensions in experimentation.

## The complexity work of methylation arrays

Biomarkers are molecular entities that can play disparate functions in epigenetics. In the most straightforward sense, they are descriptive biological differences that contribute to identifying risks of developing a disease, or offer signatures for monitoring such conditions. Specific epigenetic biomarkers have been used to assess the efficacy of psychotherapy treatment (Vinkers et al., 2021), or to predict the intensity of symptoms in persons affected by stress-related conditions (e.g. PTSD) (Vukojevic et al., 2020). Epigenetic biomarkers can also play a mechanistic function. In this sense, they are often part of an explanation—as components of a physiological process—concerning how chronic/acute stress can result in the development of psychopathologies (Chalfun et al., 2021). Stated otherwise, epigenetic biomarkers are often narrated as the material substrates of the biosocial complexity of stress-related disorders. In this section, I focus on the role that common approaches to biomarker discovery play in nourishing specific representations of biosocial processes producing health effects. How do scientists isolate the biological substrates of stressful experiences? With what commitments to biosocial complexit*ies* enabled by the ordinary tools at their disposal? I will illustrate this point by focusing on the most studied epigenetic marker—in general and among the project’s informants—which is DNA methylation.^9^ Sandra, a social epidemiologist, tells me:

We tend to use methylation because it is easy to measure. Most studies use arrays that are easy to use with large samples. But we know this is not sufficient; for instance, we do not even have read-outs of the functional effects of methylation patterns. So, we don’t really know, besides animal evidence, whether this has an impact.

DNA methylation involves the chemical addition of methyl groups in between a C (cytosine) and a G (guanine) of a DNA sequence (henceforth CpG), with effects on gene regulation and transcription. A CpG located in the promoter region of a gene will tend to promote expression when de-methylated or to inhibit it when methylated. While methylation at the level of single CpGs is a discrete difference (i.e. a CpG is methylated or not), most of its measures are provided and compared in quantitative percentages. This is due to the relatively heterogeneous composition of experimental samples (not all methylation sites mapped belong to the cell type or to the allele of interest). In a typical study design, the average methylation of each mapped CpG is compared to a control to identify statistically significant differentially methylated positions (DMPs) or regions (DMRs) correlating with the phenotype under study. Many informants reported that these associations mean that exposures can change methylation during the life course, although others underline that methylation patterns can only change in development or due to individual genetic differences. Sandra’s words underscore the difficulty—shared by many—of disentangling the non-linear relation between an exposure, a methylation mark, and a phenotype of interest in association studies of DMPs/DMRs. A single locus of the genome (a single CpG, or more likely a region of contiguous CpGs) is taken to be the biological mechanism that joins a stressful experience with the phenotype under study. However, this approach ignores that these differences may be due to other connected loci, regulatory networks established in development, functional effects of individual genetic differences, other exposures, timing, or a combination of these elements (in addition to stochasticity). Primary evidence for the way methylation studies simplify biosocial processes can thus be found in the tools that Sandra and other scientists employ for this purpose. To map DNA methylation modifications, researchers rely on specialized technologies, such as the methylation arrays commercialized by the company Illumina. These arrays have made researchers very prolific. As another informant puts it: ‘they made us good at generating data, but that’s all we did’ (Interview Joanna, Molecular Toxicologist). Yet, this widespread attitude ignores how these arrays constrain the resulting representations of social-to-biological transitions producing disease.

First, the arrays do not map all genomic regions. While theoretically possible through genome sequencing, doing so is often practically impossible because sequencing would be too expensive for most studies with large sample sizes. Arrays are a relatively cheap alternative which enables scientists to measure DMPs on a series of pre-set probes distributed across the genome. From the 27K assay developed in the late 2000s, which could investigate approximately 27,000 CpGs located across 15,000 genes, state-of-the-art technology (at the time of data collection for this article) has moved to the analysis of approximately 850,000 CpGs. While this coverage is a major expansion of analytical capacity, it represents only CpGs that are located around protein-coding genes and enhancers, meaning 3% of all potentially methylated cytosines in a genome (Riancho et al., 2016). In the words of a genomic scientist that contributed to developing these techniques in the early 2000s: the biology of how our genome responds to the environment (through epigenetic modifications) ‘is a compromise; a good one, but a compromise’ (Interview Hans, Genomic Scientists). This prompts two questions: can methylation of certain genetic regions alone explain these complex biological processes just at the level of the genome? And what do these probes relegate to the background concerning the epi-genomics of stress? Producing biosocial complexity in epigenetics works under the assumption that methylation of certain genetic regions alone can explain the genome-wide biology of stress response. But the biology amenable to these tools is only that of the few pre-established probes ‘sparsely’ covered by the Illumina arrays (Yehuda & Flory, 2018, p. 492).

Second, methylation arrays are skewed towards a specific understanding of the functional role of these epigenetic markers. These methods are a direct derivation of genome sequencing techniques: the arrays process DNA that has been chemically treated with bisulfite for the purpose of epigenetic analysis. This operation converts unmethylated cytosines to uracil (a nucleotide normally not present in DNA, but in RNA), while leaving methylated cytosines unaffected. Patterns of methylation can thus be gleaned by reading the DNA sequence after bisulfite treatment. This means that arrays are nothing more than a snapshot of the specific methylation status of a cell’s DNA at a specific time point. They are a static measure of a virtually dynamic, multi-layered and spatialized process of cellular (let alone organismic) reaction to stress, which happens only partly as chemical changes in individual CpGs. These tools orient scientists’ attention to a specific level of genome function (chemical modifications of DNA sequences), which leaves out many others: chromatin modifications, topographical rearrangements, and RNAs, to name a few. These methods do not reveal much about the circuitry of a cell affected by exposures and its physiological relevance, especially in studies that associate one specific epigenetic change with a phenotype. Rather, they perpetuate a view of biological differences as being confined to sequence-based information. Since chemical modifications of DNA sequences are the only level of biological regulation studied by arrays, the result is that these biological differences appear as the only ones making a difference in biosocial processes of health differentiation. More than a technique to dissect the fine-tuned biology of stress or even of the epigenome, arrays are geared towards reducing multi-level processes to modifications in single CpGs. One major technical constraint on the study of stress-related exposures is that these tools do not measure an underlying genomic—let alone organismal—response to stress. They merely measure the state of one biological parameter (i.e. methylation of candidate CpGs), which scientists have come to see as a potentially relevant biomarker for these conditions.

Sandra’s words also hint at the reasons why scientists are invested in these discrete markers as *the* material substrates of stress embodiment. Animal studies and the scaffolding of biological homologies between rodents and humans play a key role (Nelson, 2018). As an example, a neuroscientist named Joseph informed me about one of the highly studied methylation loci in the epigenetics of stress: the promoter 1_F_ of the glucocorticoid receptor gene NR3C1 (GR gene). 1_F_ is one of the nine known variants of the first untranslated exon of the GR gene in humans. Methylation differences in this portion of DNA have been reported in several observational studies in humans, to the extent that some consider it a promising biomarker for clinical use (Chalfun et al., 2021). Originally, Joseph argues, the interest in GR surged from an influential study in rodents where DMRs in this gene were associated with the social environment in early life (Weaver et al., 2004).^10^ This type of animal research delineated the role of rodent GR gene exon 1_7_ in animal brain physiology and adult behaviour, and then retrofitted the relevance of this mechanism for the homologous 1_F_ exon of the human GR gene. Joseph mentions a review co-authored by the senior author of this study where the authors argue that there exists a ‘compelling consensus’ on the homologous mechanisms regulating the physiology of stress response in rodents and humans (Turecki & Meaney, 2016, p. 91). Yet, Joseph asks: what makes us think that the function of exons 1_F_ and 1_7_ is the same in the brain of a human and a rodent? Joseph points out that most research explores the postulated homology between exon 1_F_ and exon 1_7_, and only a few of the remaining ten exons have been tested for methylation differences. More importantly, Joseph’s comments reveal that making interspecies homologies is a translational endeavor of its own kind, one that tames the limitations of these studies and works out a manageable complexity of stress biology. This goes as follows: exposures yield methylation patterns in distinguishable genetic regions, which in turn yield behavioural phenotypes.

Yet, stress may not, according to Joseph, fit the regularities and linearities postulated by these homological representations:

The inconsistencies, I am afraid, are there in the face of consensus. The truth of the matter is that the same mechanism in humans presents small effect sizes and explains only 2% of the symptoms in the population. Plus, we should not ignore what people actually observe: it’s hypomethylated in mouse, it’s hypermethylated in humans, or it is hypermethylated in blood and not in saliva. Plus, for many of these marks, we don’t know whether it’s a risk, a consequence of pathology, a consequence of the stressful event, or some perinatal effect, or just pure genetics.

**Figure 3.**
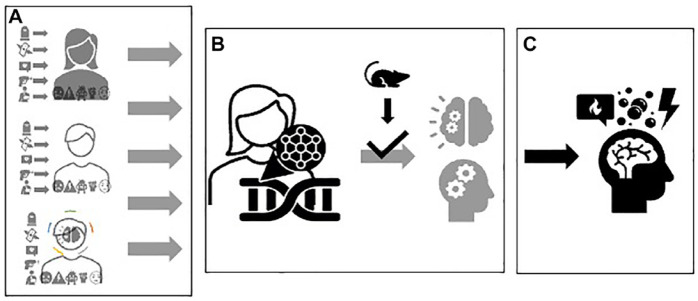
A linear and mechanistic representation of the biology of stress. Regardless of their connection with views and instruments measuring psychosocial stress (box A), tools such as DNA methylation arrays enable a linear and mechanistic type of complexity work around the biology of stress. Here, this biology is reduced to the static measure of one sequence-based information (i.e. methylation), supposedly representing organismal response to stress based on homologies drawn from animal research (box B). This information can then be used as biomarker in correlation to a known disease phenotype (box C).

Producing a coherent representation of how ‘the social’ becomes ‘the biological’ and how it loops back into patterns of behaviour or psychopathology, rests upon research designs, tools, methods, and assumptions (e.g. interspecies homologies) that constrain these representations (see Rheinberger, 2010). The archetypal version of this complexity work uses the know-how and technological repertoire of genomics. This consists of interrogating sequence information in CpG dinucleotides. In doing so, scientists employ the cheap option of arrays because of the cost limitations of full genome sequencing, or the risk of being unable to produce statistically significant knowledge worth publication, as well as the opportunity arrays offer to accumulate data on previously unknown associations. Yet, arrays also refract, or inter-mediate an ensuing version of the biology of (psycho-social) exposures. This complexity work (Figure 3) draws attention to *a linear, mechanistic representation* of how experiences associate with molecular/pathological alterations (few differences in methylation), neural dysfunction and behavioural patterns. This assemblage simultaneously relegates multi-dimensional understandings of this process to the background, and it avoids venturing into a read-out of these molecular events in their connection with cellular, tissue, physiological, and organismic processes. To highlight the pathological relevance of these observations, a whole different epistemic process is needed, one which postulates that these biological alterations in humans and animal models have homologous physiological and behavioural effects. The complexity work of methylation arrays (directed at the discovery of epigenetic biomarkers) depends therefore on an assumed continuity of neural functioning across species, more than on a clear knowledge of these biological processes and their contribution to the embodiment of stressful experiences.

## Entangling stress: ‘healthy’ brain, resilience and pathophysiology

As Joseph’s comments suggest, some actors express the need to move beyond the established research designs of association studies based on methylation. This section explores how these critical assessments are enacted as what I call practices of complexification.

Joseph is the main author of an article widely cited as one of the first studies complementing measures of DNA methylation in exon 1_F_ of the GR gene with functional analyses in different tissues. Specifically, these included an analysis of GR expression in peripheral samples (brain tissue being inaccessible), as well as memory tests in two human cohorts. As he carefully explained, this experimental configuration was only a minimal deviation from the standard design of association studies in epigenetics. The deviation was minimal, he argued, because of considerations of feasibility and budget. Yet, he maintained that it is important to underscore how it allowed them to recast biomarkers such as methylation status of GR.

His article compares differences in median and interindividual variability of GR-1_F_ methylation between a group of individuals affected by PTSD and a group of healthy individuals. These variations, the article shows, largely overlap. Thus, Joseph asked: if methylation values (and their variance) among those exposed to trauma resemble those of the unexposed, what is the causal relationship between the acute stressor and the observed differences in methylation? The fact that these same variations could be observed in a healthy population, Joseph claimed, means that differences in methylation are not traces of trauma. Rather, these differences pre-exist traumatic events. In contrast to the view portrayed in the previous section, GR-1_F_ appears here as a biological risk factor for the disorder, which is likely due to a combination of early-life programming, genetics, or even stochasticity, and constitutes a vulnerability to disease in the face of traumatic exposures. Furthermore, Joseph’s article also tested memory functioning among the PTSD patients and the healthy group—since ‘it is well known,’ I learn, that ‘glucocorticoids [whose availability in the brain is affected by GR activity] have a role in memory’ (Interview Joseph; Neuroscientist). An association was found between GR-1_F_ methylation and lower intrusive memory (in the PTSD group) or picture-recognition memory (in the healthy group). This ‘shows,’ he maintains, that the ‘effects of this biological modification operate preferentially through memory,’ and that ‘these subtle epigenetic modifications are at play both in psychopathology and in normal functioning of the brain.’ In Joseph’s article, timing, life trajectories (e.g. early-life experiences), the memory phase affected, as well as glucocorticoids in the brain (due to GR gene expression) all modulate stress-related symptomatology in the face of exposure to trauma.

Stated in the terms of complexification, Joseph presents an alternative research design and use of the tools of epigenetics, which configures an alternative representation of acute stress, GR-1_F_ methylation, neural processes, and disease from the one we encountered above. Here, this relationship is *made* as a ‘complex web of interactions’ where ‘timing or temporal sequences of events’ matter to make sense of biological differences (Interview Joseph). More than a signature biology (pathology) of stress-related diseases, epigenetic modifications highlight—within *this* network of material-semiotic relations—that the border between stress physiology and pathology is ‘not fully understood’ (Interview Joseph). As a different type of complexity work, Joseph’s version of the epigenetics of stress-related conditions produces a less linear representation of these biosocial processes. Trauma does not flow from exposure to altered epigenetic patterning, which in turn leads to pathology. Rather, his work rearranges the design and combination of tools in epigenetics to produce another understanding of GR gene methylation as biomarker of traumatic memories and PTSD. The biology of GR is here inseparable from life trajectories (i.e. developmental processes); it is inseparable from higher functions of memory through the role of glucocorticoids levels (as affected by GR); glucocorticoid levels are inseparable from genetic differences and, again, developmental effects. In Joseph’s setting, multiple courses of action, with different biological and psycho-social origins, orchestrate the physiology and pathology of stress. Joseph’s mode of producing biosocial complexity uses ideas and tools to show that a mere correlational biology of exposures does not suffice to capture the multiple social-to-biological transitions that produce (psycho) pathology. This, again, does not mean that he succeeds in making the most of these complex biosocial processes, nor does he pretend that this is the case: ‘We are merely scratching the surface,’ he laconically concludes (Interview Joseph). Yet, through the experimental configuration he chooses, Joseph detects stress differently from the simplistic association of harmful experiences with molecular alterations described above. Is Joseph’s enactment of a different biosocial complexity a trivial matter? Once we attend to what this concrete sociotechnical work *does*, one can notice how this study complexifies the imbrication of biosocial processes in coping with stress we encountered in the previous section.

During an online consortium meeting, another informant, Andreas, hammered home a similar point about the importance of considering a stress response as something ‘good’ or ‘healthy’ that sometimes ‘goes awry.’ Andreas is a basic researcher whose group uses a mouse model of acute stress. As he explained during his talk, in ‘most cases we are facing stressors in everyday life that we can deal with perfectly fine.’ In their model, Andreas and colleagues can see that ‘though the procedure is very stressful’ the ‘day after the animals are completely fine as they do not show any behavioural difference’ compared to non-stressed animals. In Andreas’ view, stress is mostly something that the animals ‘for all intents and purposes’ can overcome. ‘Our job,’ he concludes, has to be ‘chasing the healthy response to stress as it unfolds onto different waves of molecular events over time: and I am drawing your attention here to the “over time”’ (Fieldwork notes, consortium meeting).

Andreas’s lab has a growing track record on the characterization of the cascade of molecular processes that make up stress-induced responses. Though still employed by his lab, epigenetic methods do not represent the sole technique for the stratification of the molecular subcomponents of stress. As an example, Andreas’s lab uses phosphoproteomics, which is a recent technique for detecting a chemical post-translational modification of proteins that changes their structure and thus modifies their function. Protein phosphorylation, I learn, is a well-established signal for the stress response of eukaryotic cells, which it is possible to measure thanks to technical advances in mass spectrometry and related bioinformatic resources. As explained in one of their publications, this is the quickest molecular change one can detect and so, as expected, data from Andreas’s lab have shown that minutes after a stressful event, one observes a strong wave of protein phosphorylation changes that wane in thirty to forty-five minutes. Epigenomic or transcriptomic modifications operate on different temporal frame: while transcription changes can be detected a few minutes after the acute stress, the epigenetic modifications of gene expression operate at minimum within hours following the stressful event. Andreas underlined in his presentation that these multiple levels of biological activity will alter regulation and transcription ‘in a temporally-defined manner’ (Fieldwork notes, consortium meeting) and in a consistently similar way across several types of acute stressors—as confirmed by *in vivo* data of his group. Stress response is a tightly ‘choregraphed dance’ that has a beginning and an end, claimed Andreas. As he summarised it: ‘we know what should be turned on and when at the peak of the response and in its time window.’ The idea is then to use ‘stronger or long-lasting stressors to screen for points of deviation’ from the pattern detected in the case of the ‘healthy’ stress response. The biological states associated with psychopathology are, according to Andreas and his group, one and the same with what several call ‘stress resilience’ (Richter-Levin & Sandi, 2021).

**Figure 4.**
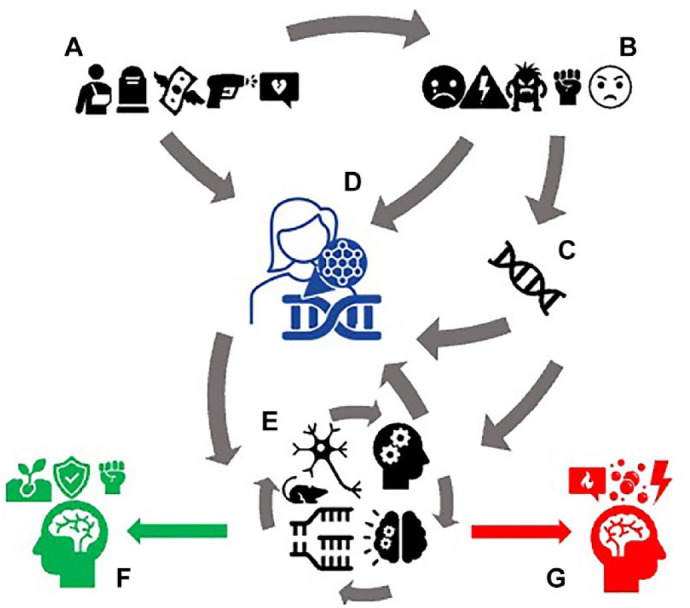
The assemblage of a dynamic and multi-layered view of stress. Multiple courses of actions and looping effects, with different biological and psychosocial origins, orchestrate the body’s response to stress. So-called stressors (icons in group A) and stress responses (group B) interact with one another, or directly affect the epigenome (group D) with/without the contribution of individual genetic differences (group C). This physiological response to stress can be studied in animals, can be mapped in different tissues, regulatory networks or functional measures (group E) and feeds back into cell functioning through the epigenome (among other mechanisms). These molecular and physiological processes can lead to both healthy stress coping (resilience) (group F) and stress-related pathophysiology (group G).

Similar to Joseph, the point for Andreas and his group is to use their laboratory practice to produce a different view of the complexity of stress-related physiology and pathology. Their suggestion is that healthy processes of stress homeostasis, reversible and timely epigenetic and/or proteomic modifications are all one and the same with the molecular processes of chronic stress or allostatic load leading to pathology. To further this view (Figure 4), Andreas and his colleagues practice multi-omic methods. During the plenary panel discussion at the consortium meeting, the chairman posed a question to all presenters: ‘what would be “the” marker of stress across rodents and humans for translational validation?’ ‘I was hoping for my connection to break down before this question,’ joked Andreas. After a puzzled round of answers—in which every speaker reiterated the importance of their own contribution—one of the coordinators of the consortium jumped into the discussion. ‘The field is moving beyond the idea of one marker: it is likely that our prospect is rather to embrace the idea of a multi-layered, multiple marker assessment … a score, at best, which may help navigate how diverse in susceptibility and disease we are’ (Fieldwork notes, consortium meeting).

As we have seen in the case of Joseph, another overlapping (and declaredly multi-level and dynamic) view of biosocial complexity could also be generated with different tools and configurations of research. The point I wish to underline is not that these experimental systems succeed in escaping the simplification of life’s complexity in biological experimentation, or in capturing all the allegedly essential characteristics of a dynamic representation of these biosocial processes. Rather, my point is one of multiplicity (Law & Mol, 2002): these researchers enact distinct complexifications of the biology of stress through distinct technical work, not merely through rhetoric. In doing so, they enact different realities for these biosocial processes. What’s in it for social studies of postgenomics and complexity?

## Discussion

This article has explored a concern with managing complexity in experimentation in postgenomics. It delineates scientists’ strategies for navigating tensions between reductionism and holism, or simplicity and complexity, in epigenetic research on stress. This reveals that different arrangements of laboratory techniques (e.g. methylation arrays, functional neuroscience methods, multi-omic analyses), study designs (e.g. the inclusion/exclusion of functional analyses) and methods to apprehend stressful experiences (e.g. questionnaires, measures of stress, scores of BPS complexity) assemble different representations of stress as a complex biosocial process in postgenomic research. Specifically, my argument is that biosocial complexity is neither dropped from epistemic practices to become talk about unfinished *post*-genomic science, nor it is simply used instrumentally or pragmatically to secure a new-fangled legitimacy for post-*genomic* science. Rather, complexity is enacted differently through scientists’ technoscientific ambitions and practical engagements. In the case of epigenetics, an established form of complexity work is that of generating biological homologies between animals and humans, which grounds the identification of putative stress-induced biomarkers in the methylome. This form of complexity work rests heavily on the possibility of objectivizing biological and psychosocial stress through tools (e.g. methylation arrays) that capture stressors and/or responses to stress. Yet, not all complexities are the same within these scientific practices. Some actors deem these forms of complexity work too linear, simplistic or hierarchical. Thus, their acknowledged triviality inspires a complementary kind of epistemic work, which I have qualified as complexification. This entails a methodical and methodological adjustment of these experimental systems, an element of ‘play and craft’ (Levin, 2014, p. 569) that is meant to apprehend stress differently. For instance, the biology of stress can be reinterpreted within the context of patient’s psychosocial co-morbidities, or as multi-dimensional, temporally expanded, biological and/or psychosocial phenomena.

What I call complexification is an alternative tuning of molecular biology’s experimental systems, which is in full continuity with what many (both actors and critics) consider a simplistic form of complexity work. Accordingly, one could focus on how little these practices diverge from one another, dismissing them as (yet another) reduction or simplification of complexity. To reiterate why: they are reductive because ‘they order, divide, simplify, and exclude’ certain bits of biosocial processes to highlight others (Law & Mol, 2002, p. 2); they are simplistic because they can hardly be reconciled with the thick ontologies of the biosocial in social and human sciences (Bieler & Niewöhner, 2018; Niewöhner & Lock, 2018). Yet, the main point of this article has been to approach these practices beyond such clear-cut dichotomies of reductionism and holism, or simplicity and complexity. Following a thread of STS scholarship (Law & Mol, 2002; Nelson, 2018) and philosophy of science on complexity (Rheinberger, 1997), I have focused on practices and the multiple representations of complex biosocial processes they enact. Such a sociology of complexification has the primary objective to contribute a renewed emphasis on material assemblages, experimental practices, and epistemic representations to STS studies of complexity and of postgenomics. In a nutshell, a study of complexification sets out to describe the multiplicity of complexity work in science. Mapping these complexit*ies* is a crucial task not solely for the sake of making sense of them (Law & Mol, 2002). Rather, it is a way to highlight the productive features of different kinds of complexity work as well as their shortcomings (see Mann & Chiapperino, 2023). In this respect, studying complexification complements the existing attention in STS on (1) complexity as mere discursive/rhetorical device to explain away the knowledge gaps of a scientific field (Arribas-Ayllon et al., 2010; Panofsky, 2015); (2) complexity as tool to establish expertise and normalize methods (Dan-Cohen, 2016); (3) complexity as pragmatic enactment guided by considerations of opportunity and feasibility (Niewöhner, 2011). Through the lens of complexification practices, complexity may not be simply understood as the ‘gap between our knowledge and some notion of biological systems,’ or the ‘reality’ out there (Dan-Cohen, 2016, p. 908). Rather, complexification suggests looking into the multiple relations between tools and explanations, concepts and methods, experimental designs and knowledge, which emerge at the crossroads of often-irreconcilable epistemological inclinations in science.

Finally, this attention to the panoply of complexit*ies* in a scientific field can also be useful for probing its policy potential. Epigenetics has already attracted a lot of interest for its potential value for action in relation to the health consequences of environmental exposures and the embodiment of inequalities (Dupras et al., 2019). Yet, the actionability of this knowledge for such purposes is not clear. Interindividual epigenetic variation can hardly be connected to the intricate social or environmental processes that produced it. In other words, this field exemplifies how the complexity work of biomedical sciences hardly matches the complexity politics required to address these challenges in policymaking (Wagenaar & Prainsack, 2021). Complexification offers an analytical tool to parse the different practical affordances of biosocial complexit*ies* enacted in experimentation. Some of the practices I investigated offer a linear view of epigenetic modifications as biological proxies of social conditions, lifestyles and/or environmental exposures. In this respect, they could bolster the idea that these individual differences are all that matter for public health action on stressful environments. Other attempts are foreign to and defy the conventional wisdom of ‘the’ biomarker of a stressor in epigenetics. These researchers frame stress as a multi-dimensional and temporally distributed biopsychosocial dynamics. Still others postulate that stress as a biopsychosocial process in epigenetics straddles the normal and the pathological. These attempts deserve scrutiny beyond their role as simplification devices, but as resources for public discourses, policymaking, and public health interventions into the biosocial complexity of our health. Is the knowledge base offered by these representations of stress a concrete opportunity to differently interrogate existing policy claims addressing health and environmental inequalities? If ‘deleting the complexity of “social environments” [in epigenetics] has sociopolitical implications’ (Louvel, 2020, p. 6), then how crucial is it to interrogate the political undertones of the opposite movement I qualified as complexification? Can the socio-political circulation of *this* knowledge about biosocial complexity avoid established critiques of biosocial facts in epigenetics? Can dynamic views of stress—insisting on resilience and reversibility—replace simplistic molecular explanations in informing action on intricate social-biological processes of health differentiation? These are just a few of the questions that a study of complexification brings to the fore for future political scrutiny of postgenomic science in society.

## Notes

1. There have been times complexity was used in my fieldwork as a synonym for ‘complicated’: that is, to refer to what is incomprehensible, difficult to disentangle, or not amenable to straightforward prediction. Often, however, complexity designated the characteristics of the system under study: the epigenome, and the organism writ large, as multi-level entities highly sensitive to all kinds of influences and, notably, psychosocial stress. These uses of the term resonate with notions of organismal complexity put forward by developmental systems perspectives on the genome (Oyama, 2000, 1989) and, more recently, on the epigenome (Lickliter & Witherington, 2017; Moore, 2017; Witherington & Lickliter, 2017). Within this tradition, the notion of complexity relates to productivist and constructionist views of organism-environment relationships (Lewontin, 2001, pp. 48–57) whereby the two are temporally and spatially entangled in ways that make causality of life processes happen at multiple levels and from multiple directions.

2. Niewöhner (2011), and others (Chiapperino, 2021; Penkler, 2022), have shown that views of biosocial complexity exist among epigenetic scientists, although they are often untheorized and divergent.

3. Further inquiries are needed into practices in countries and places beyond this selection to produce a pluralistic view of complexity in laboratory practices of epigenetics on a global scale.

4. See Young’s (1997) *The Harmony of Illusions* for a historical and ethnographic reconstruction of how the category of PTSD emerged in the treatment of Vietnam veterans.

5. For the sake of de-identification, the references cited illustrate the diversity of approaches among my informants without necessarily being their work.

6. I will not expand here on the highly choreographed activities that produce stressful experiences in settings of epigenetic research involving non-human animals. These have been analyzed elsewhere (e.g. Chiapperino, 2021; Lappé, 2018; Niewöhner, 2011).

7. COMPRECARE is a pseudonym used for de-identification. Using the actual name of the questionnaire would have made the informants’ work easily recognizable.

8. It goes beyond the scope of this article to interrogate the distance between Bertil’s operationalization of Engel’s biopsychosocial model and its original formulations. It suffices to say here that both share a view of ‘stress’ as an exemplary case study (Engel, 1977, p. 134) to question a dualistic view of the biological and the psychosocial in medicine.

9. As Deichmann (2015) and Landecker (2016) have shown on different grounds, the metaphors, theories and practices associated with, for instance, chromatin research in epigenetics, may be thoroughly different from those associated with studies of DNA methylation. In line with the argument I am developing, one can only encourage further scrutiny of the specific representations of biosocial processes grounded on chromatin research, or any other biosocial interface for that matter (e.g. the microbiome, the metabolome, etc.).

10. This article has not only established an influential research design of association studies in epigenetics, but has received considerable attention in STS (see Chiapperino & Panese, 2018; Kenney & Müller, 2017; Louvel 2020; Niewöhner, 2011; Niewöhner & Lock, 2018).
